# An artificial photosynthesis anode electrode composed of a nanoparticulate photocatalyst film in a visible light responsive GaN-ZnO solid solution system

**DOI:** 10.1038/srep35593

**Published:** 2016-10-19

**Authors:** Yoshihiko Imanaka, Toshihisa Anazawa, Toshio Manabe, Hideyuki Amada, Sachio Ido, Fumiaki Kumasaka, Naoki Awaji, Gabriel Sánchez-Santolino, Ryo Ishikawa, Yuichi Ikuhara

**Affiliations:** 1 Fujitsu Laboratories, Ltd., 10-1 Morinosato-Wakamiya, Atsugi, Kanagawa, 2430197, Japan; 2 Institute of Engineering Innovation, The University of Tokyo, Yayoi 2-11-16, Tokyo, 113-8656, Japan

## Abstract

The artificial photosynthesis technology known as the Honda-Fujishima effect, which produces oxygen and hydrogen or organic energy from sunlight, water, and carbon dioxide, is an effective energy and environmental technology. The key component for the higher efficiency of this reaction system is the anode electrode, generally composed of a photocatalyst formed on a glass substrate from electrically conductive fluorine-doped tin oxide (FTO). To obtain a highly efficient electrode, a dense film composed of a nanoparticulate visible light responsive photocatalyst that usually has a complicated multi-element composition needs to be deposited and adhered onto the FTO. In this study, we discovered a method for controlling the electronic structure of a film by controlling the aerosol-type nanoparticle deposition (NPD) condition and thereby forming films of materials with a band gap smaller than that of the prepared raw material powder, and we succeeded in extracting a higher current from the anode electrode. As a result, we confirmed that a current approximately 100 times larger than those produced by conventional processes could be obtained using the same material. This effect can be expected not only from the materials discussed (GaN-ZnO) in this paper but also from any photocatalyst, particularly materials of solid solution compositions.

Technology for producing storable energy (e.g., hydrogen, methanol, or methane) from water and carbon dioxide by irradiating a photocatalyst with sunlight is also referred to as artificial photosynthesis and is attracting attention as a promising technological solution to environmental and energy issues^1^. Various studies on visible light responsive photocatalysts with a narrow band gap have been conducted^2^,^3^,^4^. Artificial photosynthesis systems using a photocatalyst are generally classified into two types: powder-type and electrode-type systems^5^. In the former, the photoreaction occurs on photocatalyst powder dispersed in water. The whole system is inexpensive but requires an additional gas separation process because the hydrogen and oxygen are concurrently produced by a photoreaction. The electrode-type system is based on the Honda-Fujishima effect^6^, which produces oxygen and hydrogen from anode and cathode electrodes installed in a water tank by irradiating the anode electrode containing a photocatalyst with light. Because the formed gases are produced separately in the electrode-type system, the process is attractive from an industrial standpoint. As the key component that determines the efficiency of the electrode-type artificial photosynthesis, the anode electrode, which is usually formed on a fluorine-doped tin oxide (FTO) substrate, is extremely important. It requires both that the photocatalyst material be formed on an electrically conductive carrier for charge transport and that the electrode structure be highly reactive with water.

In the anode electrode, the following electron charge processes should be controlled efficiently: charge generation (the generation of holes and electrons by solar energy), charge separation (the prevention of the re-combination of holes and electrons), and charge reaction & transport (water oxidation reaction by hole & electron transport to the cathode). Because electrons and holes are generated by a photoreaction on the surface of the material through the water, a larger surface area of the material exposed to water produces a larger amount of current. Thus, smooth-surface films with their small surface areas, such as sputter and pulsed laser deposited (PLD) films, are not suitable for use in anode electrodes. Forming a nano-level particulate photocatalyst film with a large surface area is desirable for the electrode. It is not, however, easy to form a highly reactive photocatalyst particulate film with a nano-level diameter on an FTO substrate below a heat-resistant temperature of 600 °C while ensuring the electrical conductivity of the FTO. A particulate film is usually formed by electrophoresis or by applying a particulate suspension dispersed in a solvent to a substrate with the doctor blade method, drying the solvent components, and sintering the particulates on the substrate below a temperature of 600 °C. However, in the particulate film produced with these processes, the particles come off due to the physical resistance of the water or the release force of the gas generated on the surface or inside of the film because of the low adhesion between the particulates and between the particulates and the substrate. The nanoparticle deposition (NPD) we previously developed^7^,^8^,^9^ is capable of forming a film of a nano-ceramic particulate structure with high crystallinity at low temperature, and it can be considered to be optimal for the production of artificial photosynthesis anode electrodes. In this study, we applied NPD to a GaN-ZnO-based solid solution, which is well-known as a visible light responsive photocatalyst, and succeeded in dramatically reducing the band gap of the nanoparticulate film by manipulating the internal crystal structure from the atomic to nano-levels through the control of the NPD process conditions. Compared with films formed by conventional deposition technologies, the photocurrent from the anode electrode is increased by approximately 100 times. This NPD process is also applicable to various other photocatalysts and is of enormous usefulness in the formation of high-efficiency artificial photosynthesis anode electrodes.

## Results and Discussion

### Enhancement of photocatalytic properties using controlled NPD

**Figure 1:**
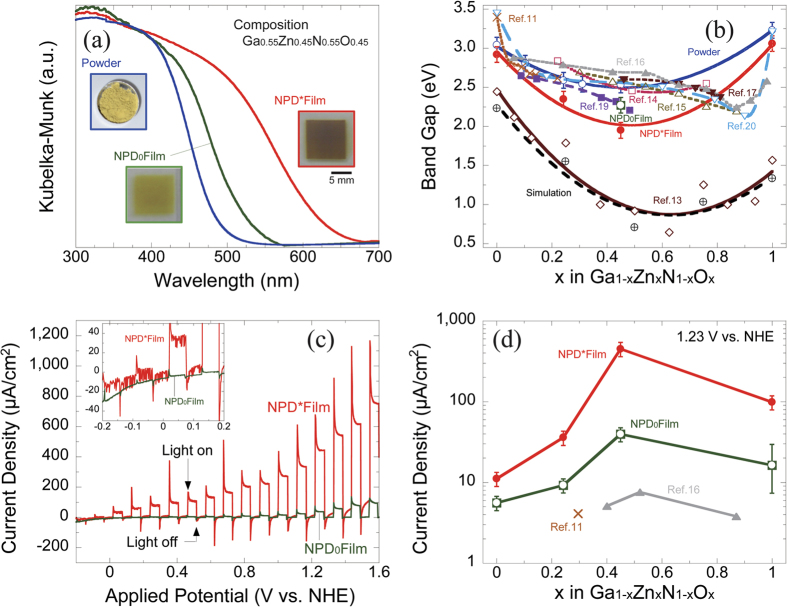
(**a**) Relationship between the Kubelka-Munk values of the raw material powder (blue), NPD_0_ film (transport velocity: 50 m/sec) (green), and NPD* film (transport velocity: 100 m/sec) (red) of Ga_1-x_Zn_x_N_1-x_O_x_ (X = 0.45) solid solution calculated from the diffuse reflectance spectra measured using a spectrometer and wavelength and their appearances. (**b**) Composition dependence of experimental values of band gaps of the raw material powder and NPD* film (transport velocity: 100 m/sec) of Ga_1-x_Zn_x_N_1-x_O_x_ solid solution used for the present research, simulation values and values already reported in the literature. (**c**) Photocurrent characteristic of the NPD film of Ga_1-x_Zn_x_N_1-x_O_x_ (X = 0.45) solid solution photo-excited by a solar simulator (under 1 SUM). The bias voltage on the horizontal axis was corrected to the reference value of NHE at a pH of 0. (**d**) Composition dependence of photocurrent characteristic of the NPD film of Ga_1-x_Zn_x_N_1-x_O_x_ solid solution (at 1.23 V) photo-excited by a solar simulator (under 1 SUM) and the results already reported. (The applied bias voltage was converted to the reference value of NHE at a pH of 0).

A GaN-ZnO-based material is a solid solution of hexagonal wurtzite structure crystals that can continuously change its composition as Ga-Zn and N-O are substituted in the same crystal structure. It is known that a GaN-ZnO solid solution has a narrower band gap than pure GaN or ZnO, and its absorption wavelength edge can be expanded to the long wavelength side as the amount of ZnO dissolved into the GaN increases^10^. Figure 1(a) shows the appearance and the Kubelka-Munk values converted from the diffuse reflectance spectrum using UV and visible light of the raw material powder of Ga_1-x_Zn_x_N_1-x_O_x_ (X = 0.45) and two types of NPD films (NPD0 and NPD*) formed on the FTO substrate using this raw material powder. The reflection absorption edge of the raw material powder is 490 nm (band gap: 2.53 eV), and the reflection absorption edge of the NPD film (NPD_0_) deposited using nitrogen carrier gas at 50 m/sec is 545 nm (band gap: 2.27 eV). The raw material powder and the NPD_0_ film assume almost the same yellow color, and a small difference can be detected in their band gap values. The NPD* film shown in the figure was deposited with a particulate transport velocity doubled from 50 m/sec. to 100 m/sec. by using helium carrier gas in the process. The film has a brown color, and its reflection absorption edge evaluated using a spectrometer was 635 nm (band gap: 1.97 eV), which is significantly smaller than that of the original raw material powder. In NPD, the ceramic particulates transported with the gas flow continuously depressurized by the vacuum pump collide and are crushed inside the nozzle. Even after that dynamic process, the crystallinity of the internal particulates is maintained, the crystal structure of only their surfaces is strained, and the surface energy of each particulate increases. The unstable crushed particulates with high surface energy re-bound through a cohesive force, and a dense ceramic nanoparticulate film is formed on the substrate at room temperature. The film can be fully sintered at a temperature approximately 500 °C lower than that for bulk ceramics with the same composition because the inside of the film is composed of nanoparticulates^8^.

Under the high transport velocity applied in this study, plasma is produced near the surface of the formed film during the deposition process. Because the material (GaN-ZnO) used exhibits piezoelectricity, it is considered to have a stronger dynamic impact than the regular NPD process, causing plasma to result from the ionization of the carrier gas by the free electrons derived from the piezoelectric phenomena of the material. In this paper, these NPD film formation process conditions were applied, but the detailed plasma phenomena are not discussed.

Figure 1(b) shows the band gap values of our synthesized raw material powder of the Ga_1-x_Zn_x_N_1-x_O_x_ solid solution and of the NPD films formed at two transport velocities of 50 and 100 m/sec from the same solid solution composition. To synthesize the Ga_1-x_Zn_x_N_1-x_O_x_ solid solution, we adopted a general solid-state reaction using oxide raw materials (Ga_2_O_3_, ZnO) at high temperature (1123 K) in an ammonia gas flow^11^. It was, however, difficult to increase the amount of ZnO dissolved in the Ga_1-x_Zn_x_N_1-x_O_x_ solid solution, and the largest amount of dissolved ZnO in our experiment was X = 0.45. When X was larger than 0.45, two phases, Ga_0.55_ Zn_0.45_N_0.55_O_0.45_ and ZnO, coexisted, and a single solid solution phase could not be obtained. The band gap of the Ga_1-x_Zn_x_N_1-x_O_x_ solid solution powder we synthesized tended to decrease with increasing ZnO content and reached its lowest value, 2.53 eV, at the solubility limit of X = 0.45 (open circle). The band gaps of the NPD films deposited under a higher velocity using this solid solution powder are marked with a filled circle. For pure GaN and ZnO, there is little difference in the band gaps between the raw material powder and the NPD film; however, the band gap of the film of a Ga_1-x_Zn_x_N_1-x_O_x_ solid solution is lower than that of the raw material powder of the same composition. This difference in the band gap increases in proportion to the ZnO content and reaches its largest value at a composition of X = 0.45, the solubility limit, and the band gap of the NPD film was approximately 1.97 eV, 22% lower than that of the raw material. A band gap smaller than 2 eV was obtained for the GaN-ZnO-based solid solution for the first time.

The Domen group used the solid reaction method to synthesize a Ga_1-x_Zn_x_N_1-x_O_x_ solid solution with a ZnO content of 30% or less and discovered that its band gap decreased as X increased and was 2.5 eV when X = 0.3^12^. Jensen *et al*. revealed in their simulation based on first-principles calculations that a concave composition dependence exists below the minimum point of the band gap at a dissolved amount of ZnO of approximately 60% and suggested that a minimum band gap value of 2.29 eV would be obtained for X = 0.525 when this simulation result was extrapolated to the experimental value^13^. This result led researchers to consider synthesis methods capable of further increasing the dissolved amount of ZnO. Syntheses under high pressure^14^, using nanoparticulate raw material^15^,^16^, using a Zn/Ga/CO_3_ layered double hydroxide^17^,^18^, and using a ZnGaO precursor^19^,^20^ were studied, and solid solutions with X = 0.8 to 0.9 were synthesized. In all of the synthesis methods, the band gap tends to decrease with the amount of ZnO added. The smallest value among the band gaps obtained thus far was 2.2 eV. The simulation results by first-principles calculations conducted by our group are also shown in Fig. 1(b). A concave curve as a function of the ZnO content with the minimum band gap at approximately X = 0.5, which is a similar result to that of Jensen, is obtained. Figure 1(c) is the photocurrent characteristic of the NPD film of the Ga_1-x_Zn_x_N_1-x_O_x_ (X = 0.45) solid solution, and (d) shows the composition dependence of the photocurrent of the NPD film consisting of a Ga_1-x_Zn_x_N_1-x_O_x_ solid solution during irradiation under the AM1.5 illumination condition (1 SUN) and literature data. All the photocurrents of the Ga_1-x_Zn_x_N_1-x_O_x_ solid solutions, including the literature data reported thus far, were converted into the value of 1.23 V of an NHE at a pH of 0 and plotted. The photocurrent characteristics of Ga_1-x_Zn_x_N_1-x_O_x_ solid solutions have been reported for films formed by the electrophoresis method^12^ and the doctor blade method^16^. The photocurrents reported thus far are on the several μA/cm^2^ level. On the other hand, the photocurrent of the GaN-ZnO solid solution NPD film with a composition of X = 0.45 formed in the present research was approximately 500 μA/cm^2^, which is approximately 100 times larger than the photocurrents of the films formed by conventional processes. By examining the internal atomic, electronic, and nanostructures of the NPD film and comparing them with those of the raw material powder, we discuss the reason why a high photocurrent characteristic can be obtained in the controlled NPD process. We also confirmed that under monochromatic light at 640 nm with a band-width of 10 nm, a photocurrent can be generated in the GaN-ZnO solid solution (X = 0.45) NPD film. The presence of O_2_ and H_2_ in a molar ratio of 1/2 after 6 hours of radiation using a solar simulator was confirmed.

### Crystal and atomic structures of photocatalytic NPD film

**Figure 2:**
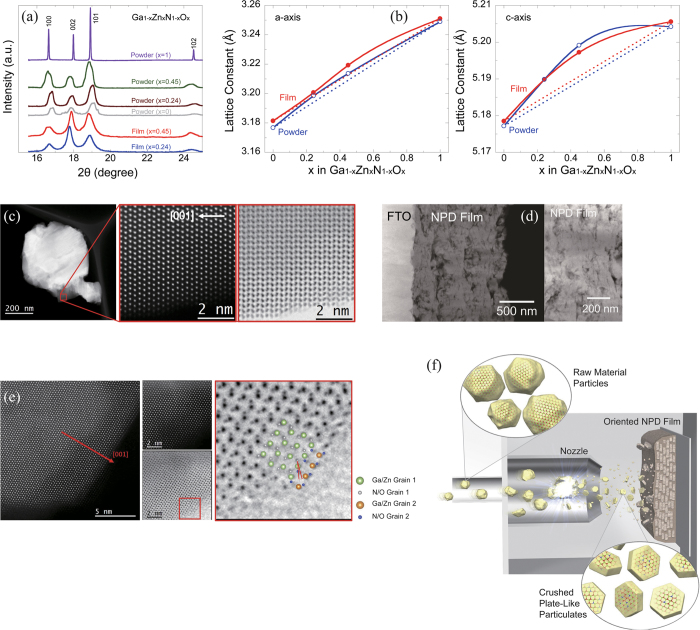
(**a**) X-ray diffraction results of the raw material powder and NPD film of Ga_1-x_Zn_x_N_1-x_O_x_ solid solution (λ = 0.8 Å). (**b**) Composition dependence of lattice constants (a-axis, c-axis) of the raw material powder and NPD film of Ga_1-x_Zn_x_N_1-x_O_x_ solid solution calculated by Rietveld analysis of X-ray diffraction results. (**c**) Low-magnification and atomic-resolution HAADF and ABF-STEM images obtained from raw material particulates of the Ga_1-x_Zn_x_N_1-x_O_x_ (X = 0.45) solid solution composition. (**d**) LAADF images of the cross-sectional-view structure of the NPD film of the Ga_1-x_Zn_x_N_1-x_O_x_ (X = 0.45) solid solution composition. (**e**) Atomic-resolution HAADF and ABF-STEM images obtained from the surface of the NPD film of the Ga_1-x_Zn_x_N_1-x_O_x_ (X = 0.45) solid solution composition. The models of the green/gray pair and the orange/blue pair are different sets with different atomic arrangement regularities. (**f**) Schematic image of the NPD process in which the oriented film of the GaN-ZnO solid solution is formed. Raw material particulates were crushed in the NPD in-line process, and plate-like crushed particulates having exposed (0001) planes were produced and accumulated with some compressive stress applied to their exposed (0001) planes parallel to the substrate, resulting in a small orientation.

Figure 2(a) shows the X-ray diffraction results (λ = 0.8 Å) of the raw material powder and the NPD film of the Ga_1-x_Zn_x_N_1-x_O_x_ solid solution. When the added ZnO is less than 45%, a single solid solution phase can be obtained, but unmixed ZnO remains in the case of compositions with more than 45% ZnO. Thus, the present study considered pure GaN, a GaN-ZnO solid solution with less than 45% ZnO, and pure ZnO, as shown in Table 1. The intensity ratio of the (0002) diffraction line of the film of each composition deposited by NPD is high compared with that of the raw material powder of each solid solution, implying that the solid solution crystals of the NPD film are oriented in this crystal plane. We studied the orientation effect in the Rietveld analysis using the March-Dollase function (MD)^21^,^22^ and confirmed that approximately 20% of all crystals were oriented in the (0002) plane, indicating a weak orientation as a whole.

Figure 2(b) shows the composition dependence of the lattice constants (a-axis and c-axis) obtained from the XRD Rietveld analysis of the raw material powder and the NPD films of the Ga_1-x_Zn_x_N_1-x_O_x_ solid solution. The a-axis lattice constant values of the raw material powder and the NPD film are almost the same and increase linearly with the dissolution of ZnO, in the same trend as the straight line connecting the lattice constant values of GaN and ZnO. The c-axis lattice also increases with increasing dissolution of ZnO, and a convex curve is drawn above the straight line connecting the lattice constants of GaN and ZnO. When the amount of dissolved ZnO in the composition is 45%, the lattice constants are the farthest away from the straight line. Past reports present similar convex curve results for GaN-ZnO-based solid solutions with an added amount of ZnO of approximately 50%^13^. This result probably occurs because the basic lattice of the GaN expands in the c-axis direction as the substituted amounts of Zn and O increase, and the balance of the interatomic distance becomes most unstable when the ZnO in the composition is approximately 50%, meaning that half of the GaN is substituted for ZnO. The c-axis lattice constant divergence of the NPD film from the straight line between the GaN and ZnO lattice constants is smaller than that of the raw material powder; this is attributable to the residual stress of the constituent particulates fixed in the NPD film.

Figure 2(c–e) show STEM micrographs of the raw material particulates and the cross-sectional-view and surface structures of the NPD films with a Ga_1-x_Zn_x_N_1-x_O_x_ (X = 0.45) solid solution composition, where we used ABF, LAADF and HAADF STEM imaging (annular bright-field and low-angle and high-angle annular dark-field). We observed various numbers of particles, and the diameter of the particulates of the solid solution is roughly estimated to be 500 nm. The shape of the particles is close enough to an isotropic, spherical shape, and the crystallinity is very high, as shown by atomic-resolution HAADF and ABF STEM images, corresponding to Fig 2(c). Figure 2(d) shows the cross-sectional-view of the NPD film formed by the powder shown in Fig. 2(c). The structure consists of particulates of various diameters ranging from several nm to 200 nm. The low-magnification LAADF-STEM image shows plate-like particulates compacted and stacked in layers parallel to the substrate. The surface free energy of GaN is the highest in the (0001) plane^23^, and the GaN-ZnO solid solution studied in this work also consists of crystals of a hexagonal, wurtzite structure similar to that of GaN and is likely to cause dislocation, meaning atomic slip on the closest-packed (0001) plane. In the NPD process, the particulates collided with one another, and slippage occurred on the (0001) face, which was exposed on the surface. As a result, the plate-like particulates exposed on the (0001) plane with a high surface energy after cracking were transported by gas, rearranged, stacked, aggregated, and cohered on the substrate or the film surface so that the energy of the whole system could become the lowest. This is the likely reason why a weak-(0002) oriented stacking structure with plate-like particles was formed. The inside of this NPD film is a non-uniform, random structure made up of particulates of different diameters, unlike that of the thin film formed by vapor-phase epitaxy. However, it is considered to be a rather suitable morphology for exploiting a high photocatalyst effect^24^. Figure 2(e) shows the atomic structure of the particulates on the outermost surface of the NPD film. In the figure, the [001] direction and the vertical (0001) face are exposed on the surface. The misalignment of the atomic arrangement was also observed inside the particulates on the (0001) face. In this case, the Ga (Zn)-N (O) bond in the third row from the surface is rotated by an additional 10 degrees from the 180-degree inverted state. A schematic image of the NPD process described above is shown in Fig. 2(f).

### Effect of electronic structure on the composition of NPD film and raw material powder

**Figure 3:**
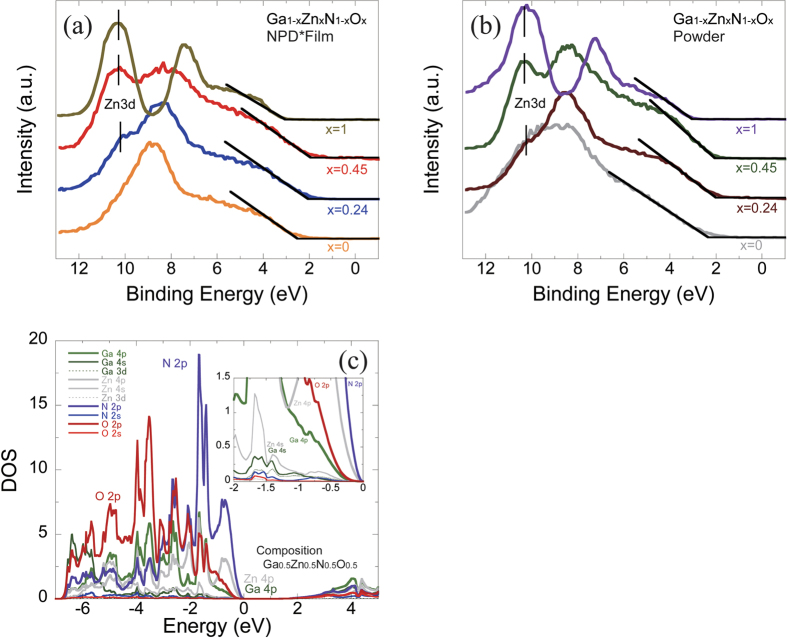
(**a**) State of the binding energy of the raw material powder observed using hard X-ray photoelectron spectroscopy (HAXPES). (**b**) State of the binding energy of the NPD film observed using hard X-ray photoelectron spectroscopy (HAXPES). (**c**) Projected density of states of the solid solution Ga_1-x_Zn_x_N_1-x_O_x_ (X = 0.5) simulated by first-principles calculations.

Figure 3(a,b) show the binding energy of the raw material powder and that of the NPD film detected using hard X-ray photoelectron spectroscopy (HAXPES). In the figures, a binding energy level of 0 was calibrated at a level of the Au 4f_7/2_ state to the Fermi level. For the same composition, there is almost no significant change in spectral behavior between the raw material powder and the NPD film. On the other hand, the spectrum is shifted to the side of low binding energy (right side of the graph) for both the raw material powder and the NPD film with increasing amount X of ZnO in the Ga_1-x_Zn_x_N_1-x_O_x_ solid solution. The spectrum end (point of intersection with the background) corresponding to a valence band maximum exists on the side of the lowest binding energy for the composition X = 0.45, indicating that the electron level of the valence band maximum of the solid solution with a ZnO amount of 45% is the highest. Figure 3(c) shows the simulated densities of states projected in the electron orbitals of the constituent elements of the solid solution of Ga_1-x_Zn_x_N_1-x_O_x_ (X = 0.5). In the simulation, the maximum valence band was regarded as the Fermi level: zero on the X-axis. The energy level combining the Zn4p, O2p, and N2p orbitals is formed just under the Fermi level, similar to a result already presented in a previous report^25^. Judging from the result of these combined orbitals, the position of the valence band likely moved to the side of high energy as the amount of ZnO increased. The conduction band minimum (CBM) consists mainly of the cation 4s and 4p unoccupied orbitals giving electrons to anions.

### Difference in radial distribution between NPD film and raw material powder

**Figure 4:**
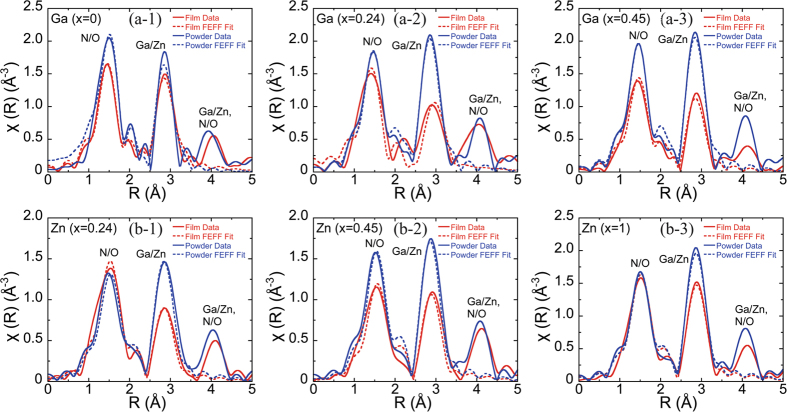
Radial distributions calculated by Fourier transformation from XAFS data on raw material particulates and the NPD film of the Ga_1-x_Zn_x_N_1-x_O_x_ (X = 0, 0.24, 0.45, 1) solid solution composition. (a-1), (a-2), (a-3): Analysis results obtained from Ga-Kedge. (b-1), (b-2), (b-3): Analysis results obtained from Zn-Kedge.

We measured the Ga K-edge (10367 eV) and Zn K-edge (9659 eV) in the XAFS of the raw material particulates and the NPD film of the Ga_1-x_Zn_x_N_1-x_O_x_ (X = 0, 0.24, 0.45, 1) solid solution composition. The XAFS χ (k) was obtained by subtracting the background from the data and standardizing the data with an edge step using Athena analysis software^26^. The radial distributions shown in Fig. 4 were obtained by the Fourier transformation of χ (k). The radial amplitude intensity reflects the order of binding. The dotted line was fitted by FEFF calculation based on the wurtzite crystal structure. It was fitted using Artemis analysis software and by introducing the shift of distance (special resolution) and the Debye-Waller structure disorder parameter σ to each group of atoms. Figure 4(a-1),(a-2) and (a-3) show the analysis results obtained for the Ga K-edge, and Fig. 4(b-1),(b-2) and (b-3) show those obtained for the Zn K-edge. Every result indicates that the intensity (amplitude) dropped at the neighboring atom peak for the NPD film compared with the powder sample, meaning that the atomic order of the NPD film decreased. This downward tendency with respect to the intensity is dependent on the composition of the solid solution. With regard to the radial distribution in terms of the Ga atoms, the intensity of the NPD film, compared with that of the raw material powder, shows a slight drop at both the first neighboring peak of N/O and the second neighboring peak of Ga/Zn for the pure GaN composition shown in Fig. 4(a-1). In the system in which 24% of ZnO was dissolved in GaN, the intensity of the NPD film considerably dropped at the second neighboring peak of Ga-(Ga/Zn) compared with the raw material powder (Fig. 4(a-2)). In the system with 45% dissolved ZnO, the intensity dropped not only at the second neighboring peak but also at the first neighboring peak Ga-(N/O) (Fig. 4(a-3)). With respect to the radial distribution in terms of the Zn atoms, the intensity of the NPD film dropped at the second neighboring peak of Zn-(Ga/Zn) for the GaN-ZnO system with 24% dissolved ZnO (Fig. 4(b-1)); in the composition with 45% dissolved ZnO, the intensity of the NPD film dropped not only at the second neighboring peak of Zn(Ga/Zn) but also at the first neighboring peak of Zn-(N/O) (Figure (b-2)). In the pure ZnO, the intensity of the raw material powder and that of the NPD film did not change at the first neighboring peak of Zn-O but dropped only at the second neighboring peak of Zn-Zn. These results confirm that in the NPD film, the interatomic distance fluctuates and the atomic order is decreased compared with the raw material powder, and the atomic order is further decreased as the substituted and dissolved amount of the Ga_1-x_Zn_x_N_1-x_O_x_ solid solution is increased. The likely reason for the decrease in the atomic order is that in the NPD process, mechanical pressure was applied to the particulates by the particulate crush occurring in the film formation process, and the atomic arrangement was consequently misaligned. In addition, as the amounts of dissolved elements of the solid solution increase in the parent body, the atomic ordering is decreased and the atomic arrangement randomness is increased. It is assumed that because the local strain due to the imbalance, that is, the difference in the ion radius, of the atomic species on the Ga and Zn sites and on the O and N sites occurring locally in the crystal structure will be the largest in the composition with 45% dissolved ZnO, the decrease in the atomic ordering caused by the mechanical compressive force applied in the NPD process was the most obvious of the decreases for all compositions.

### Effect of band gap on the interatomic distance in a crystal by simulation

**Figure 5:**
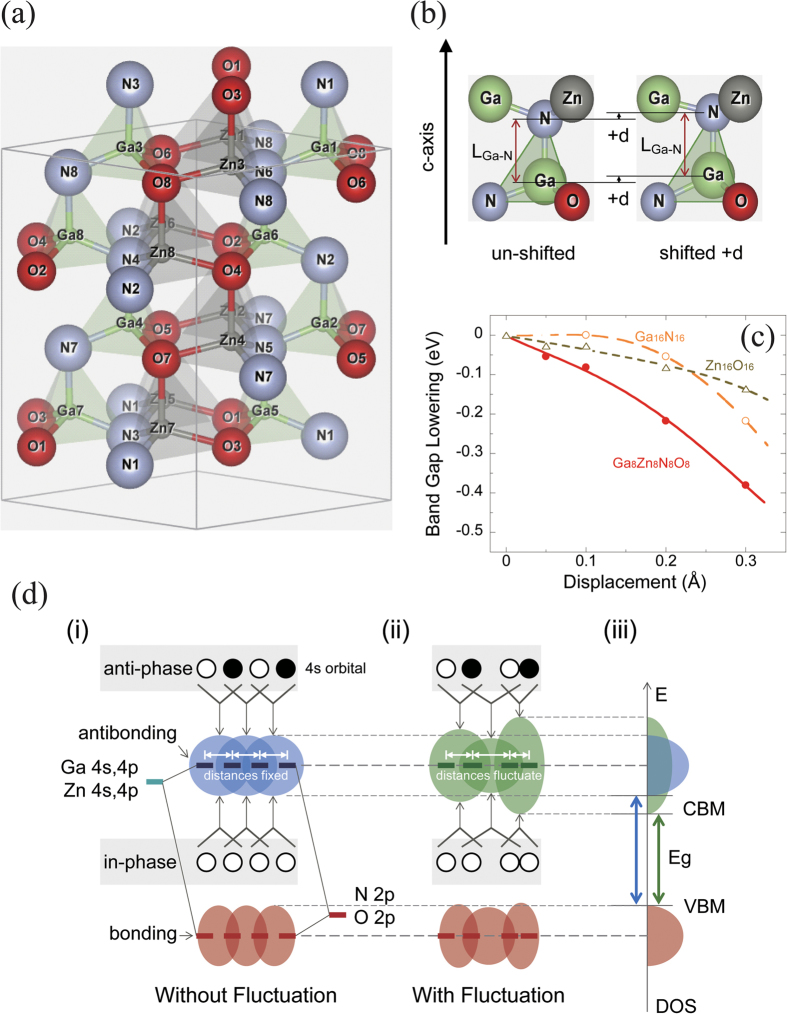
(**a**) Relaxed crystal structure model (X = 50%) of the Ga_1-x_Zn_x_N_1-x_O_x_ used for simulation. The simulation was carried out under periodic boundary conditions, and ions with the same label are completely identical. (**b**) Partial structure of an NPD film model with the displacement added to the interatomic distance of the relaxed structure. This figure shows the model viewed from the a-axis direction. Ga–N–Ga–N is positioned on the paper, and the Zn and O that are visible in the figure (Zn and O hidden behind them) are positioned on the closer side (further side). With Ga and N bonding as a pair, the displacement d changes, but the distance between Ga and N is kept constant. (**c**) Simulation results of band gap decrements of Ga_1-x_Zn_x_N_1-x_O_x_ (X = 50%), GaN and ZnO crystals at different amounts of displacement d. (**d**) Schematic diagrams explaining the decrease in the band gap energy due to the displacement of the positions of the atoms. (i) Electronic structures of the GaN-ZnO solid solution without atomic fluctuation and (ii) with atomic fluctuation, and (iii) superimposed densities of states (DOS) with and without fluctuation.

As described thus far, there was a difference in the atomic order between the raw material powder and the NPD film. In other words, the order of the atomic positions between Ga/Zn and Ga/Zn and that between Ga/Zn and O/N decreased after the NPD process. This phenomenon was considered as a factor contributing to the decrease in the band gap width after the NPD process, and we therefore carried out a simulation based on first-principles calculations and examined the effect of the fluctuation of the interatomic distance on the band gap width. Figure 5(a) shows the relaxed crystal structure model of Ga_1-x_Zn_x_N_1-x_O_x_ (X = 0.5) used for the simulation. In the simulation, a model consisting of a total of 32 atoms (ions), 16 cations (Ga^3+^ and Zn^2+^) and 16 anions (N^3−^ and Zn^2−^) was used for Ga_1-x_Zn_x_N_1-x_O_x_. The Ga_1-x_Zn_x_N_1-x_O_x_ solid solution has a hexagonal wurtzite crystal structure and is constructed with a tetrahedron consisting of anions (O and N) located at the apexes and a cation (Ga or Zn) at the center of the body. The simulation was carried out under periodic boundary conditions, and ions with the same label are completely identical, though some ions are indicated several times to draw the tetrahedron in Fig. 5(a). Both pure GaN and pure ZnO have only one anion (Ga^3+^ or Zn^2+^) and one cation (N^3−^ or O^2−^), but in the solid solution, although the composition of the whole material has a designated value, the positions of the individual ions in each ion site are random. A special quasirandom structure^13^ was adopted for the ion arrangement of the intermediate composition model to introduce the randomness of the solid solution.

To reproduce the decrease of the order, an NPD film model providing the displacement of the interatomic distance was configured based on the relaxed crystal structure shown in Fig. 5(a)^27^. Figure 5(b) shows the partial model used for the present simulation. We performed a simulation by applying the displacement d in the c-axis direction to one fixed bonding pair composed of a given cation (center in tetrahedron) and a neighboring anion (one apex in tetrahedron), with the bonding distance maintained between the cation and the anion, in the 32-atom relaxed crystal structure shown in Fig. 5(a). Because the atoms (ions) in the wurtzite crystal structure are apt to move in the c-axis direction, the displacement was applied in the c-axis direction, and the distance d was used as a variable parameter. In this model, though we changed only the distance between cations, the distance between the cation and each of the three apex anions in the tetrahedron will also be changed accordingly. However, that extent is very little because they form an angle of approximately 110 degrees with the displacement direction.

Figure 5(c) shows typical simulation results of band gap decrements of Ga_1-x_Zn_x_N_1-x_O_x_ (X = 0.5) and GaN and ZnO crystals when the traveling distance d is changed. The band gap energies of the GaN-ZnO solid solution, GaN, and ZnO decrease as the displacement increases, regardless of if the displacement is upward or downward from the relaxed structure. Because the solid solution has a multi-element (Ga, N, Zn, O) structure, the electronic interaction with other atoms is affected, and the band gap is decreased by a greater extent compared with pure GaN. When a pair of two atoms among the 32 atoms was displaced by only 0.2 Å, the band gap energy typically decreased by approximately 0.2 to 0.3 eV in this simulation. If more pairs of atoms in the 32-atom model were displaced, the band gap energy showed a further decrease. The XAFS results also show that the misalignment of the designated atomic positions of the GaN-ZnO solid solution after the NPD film formation increased compared with that of pure GaN. This is equivalent to the cases in which the degree of the decrease of the band gap relative to the displacement d was larger for the GaN-ZnO solid solution than for GaN or ZnO. Therefore, the described simulation results agree with the experimental results shown in Fig. 1(b) in that the decrement of the band gap between the raw material powder and the NPD film is larger for the GaN-ZnO solid solution than for pure GaN. The mechanism of the decrease of the band gap energy by these effects is shown in Fig. 5(d). Figure 5(d)(i–iii) shows the schematic diagrams of the electronic structure with and without atomic fluctuation in the GaN-ZnO solid solution. (i): In the normal GaN-ZnO solid solution, the Ga 4s, 4p and Zn 4 s, 4p orbitals bond directly to the N 2p and O 2p orbitals, and the Ga 4 s, 4p and Zn 4 s, 4p orbitals, which become unoccupied after the electrons move to the N 2p and O 2p orbitals, interact with each other between sites and form a conduction band. At the Γ point in the reciprocal space, the wave functions of the neighboring Ga 4s, 4p and Zn 4s, 4p orbitals are in-phase, and if the wave functions are bonding-like between them, the energy of the state reaches its lowest level, forming a CBM. At the edge of the Brillouin zone, on the other hand, the wave functions are anti-phase in the direction of the reciprocal vectors, and if the wave functions are antibonding-like, the energy of the states will become higher. These bonding-like and antibonding-like relations determine the width of the band. (ii): When displacement, resulting in the interatomic fluctuation, is applied to the distance between atoms in real space, the strength of the interaction between them and, thus, the width of the electronic states changes. The width of the state increases as the atoms become closer, and the interaction becomes stronger. (iii): If the distance between atoms changes, the states will become wider or narrower in energy. This phenomenon occurs in a local area, but the states spread spatially, so the wider part of the states will determine the band width of the whole crystal. Because the energies of the original electron orbitals without displacement remain unchanged and the bands expand, the CBM will become lower and the band gap energy will decrease.

### Dependence of the CBM and VBM of both the raw material powder and NPD film on the composition

**Figure 6:**
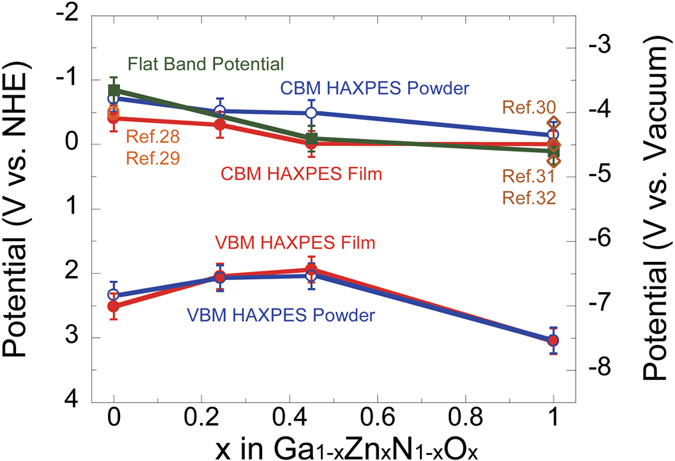
Valence band maximum (VBM) and conduction band minimum (CBM) of the raw material powder and NPD film of the Ga_1-x_Zn_x_N_1-x_O_x_ solid solution composition calculated from the results of the present research, flat band potentials calculated from the Mott-Schottky method, and values reported in past papers and the literature.

The valence band maximum (VBM) and CBM of the raw material powder and the NPD film calculated based on the above results are shown in Fig. 6. In addition, we electrochemically examined the flat band of the NPD film by the Mott-Schottky method, and the values of the pure GaN and ZnO are plotted together with the values already reported in the literature. The VBM in the figure was obtained from the HAXPES results shown in Fig. 3, and the CBM was calculated by subtracting the band gap shown in Fig. 1 from the VBM. The VBM is not significantly different between the raw material powder and the NPD film, as shown in the figure, but a composition dependence can be detected, and the VBM shows a maximum value (higher side of the graph) at 45% ZnO. This results from the effect of the combined bonding of the Zn, O, Ga, and N electron orbitals in the VBM, as described earlier. The CBM of the raw material powder decreases as the added amount of ZnO increases in the GaN-ZnO solid solution. On the other hand, the CBM of the NPD film exists on the low energy side (lower side of the graph), below the energy level of the raw material powder at the same composition. The energy level of the NPD film with 45% ZnO is slightly higher than the standard potential of hydrogen (just above the standard potential of hydrogen on the graph). The position of the flat band of the Ga_1-x_Zn_x_N_1-x_O_x_ (X = 0.45) NPD film calculated by the Mott-Schottky method is slightly higher (upper side of the graph) than the film CBM energy level calculated through HAXPES and UV-visible light spectrometry. The values of the positions of the flat bands of the pure GaN and ZnO reported in the literature^28^,^29^,^30^,^31^,^32^ are close to our measurement results. The results shown in Fig. 6 lead us to the conclusion that the NPD film of the Ga_1-x_Zn_x_N_1-x_O_x_ (X = 0.45) composition can meet the two optimal requirements (band gap and band level) of artificial photosynthesis anode electrodes.

As described thus far, we have proven that the application of the NPD process to photocatalyst materials could form films ensuring a nanoparticulate structure with a band gap smaller than that of the original materials and high crystallinity. The NPD process is considered effective, in particular, for solid solutions that allow the easy displacement and variation of the positions of the atoms. In the present research, we studied the process with GaN-ZnO, which is a representative visible light responsive photocatalyst, but the results presented in this paper are applicable to various other photocatalysts and make a great contribution to the development of high-efficiency artificial photosynthesis anode electrodes.

## Methods

### Measurement of band gap

In the measurement and evaluation of band gaps, we used an ultraviolet and visible diffuse reflectance spectrometer (V-650) by JASCO Corporation to measure the reflectance of each wavelength. The band gap after measurement was calculated using the method described below. The measured reflectance was substituted into the Kubelka-Munk function,


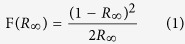


where R_∞_ is the absolute reflectance.

Equation 2 proposed by Tauc *et al*. was used to determine the band gap. We plot the data in the graph with the energy (hν) set on the horizontal axis and (hνα) ^(1/n)^ set on the vertical axis, and the intersection point of the gradient (to be precise, the gradient at an inflection point on the graph) and the baseline was calculated. The value of the intersection point on the horizontal axis was adopted as the band gap.


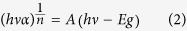


where h is the Planck constant, ν is the vibration frequency, hν is 1239.7/λ (λ: wavelength), α is the absorption factor (replaced by F(R_∞_)), Eg is the band gap, A is the constant of proportionality, and n is 1/2 in the case of a direct transition.

### NPD film formation

The aerosol-type nanoparticle deposition (NPD) equipment is composed of an aerosol generation system, a deposition chamber and a vacuum system. No heat source was placed in this deposition equipment. Raw powder of Ga_1-x_Zn_x_N_1-x_O_x_ that was more than 99% pure with an average particle size of 500 nm was set in the vessel of the aerosol-generation system and vibrated at 10 Hz. Then, 99.9% pure oxygen, nitrogen or helium with a gas pressure of 0.2 MPa was introduced into the vessel. An aerosol consisting of gas and raw powder was generated in the vessel and transferred into the nozzle. Inside of the nozzle, the raw powder was collided and fractured. As a result of the brittle deformation of the raw powder, crushed particles are generated and sprayed from the nozzle. In this fracturing process, nanoparticles exposing a new fracture plane are formed. The GaN-ZnO solid solution with a hexagonal wurtzite structure used in this study is likely to be cleaved on the (0001) plane. As a result, the particle shape tends to be changed to plate-like. (It is found from TEM observation that this phenomenon does not occur for all particles.) These sprayed plate-like particulates exposing an unstable new crystal plane are accumulated on the substrate, stabilizing it to the state of the lowest surface energy. The in-chamber base pressure was controlled to be less than 10 Pa using a mechanical booster and a vacuum pump. The substrate in the chamber was bombarded for 10 minutes with particles from the aerosol expelled from the nozzle at a gas speed ranging from 50 to 100 m sec^−1^ by changing the gas flow and type. Nitrogen (sound velocity: 337 m/sec.) and helium (970 m/sec.) were used for the carrier gases of NPD0 and NPD*, respectively. The gas speed was calculated from the gas flow rate in the area where the gas passed through the opening of the nozzle. Thus, the NPD film was deposited at room temperature. The film in this experiment is 3 microns thick. As reported in the previous paper, it is confirmed that the as-deposited film is composed of particulates with a low crystallinity, but a high crystallinity can be obtained by annealing at a temperature above approximately 500 °C^8^. In the present research, annealing was performed for 30 minutes in a nitrogen atmosphere maintained at 600 °C after the film formation, and the NPD film with recovered crystallinity was used as the sample for all evaluations.

### Synchrotron radiation measurement

High-brilliance synchrotron measurements were performed at the SPring-8 Synchrotron Radiation Facility, Hyogo, Japan. Two beamlines of the SUNBEAM Consortium, the 16XU undulator beamline and the 16B2 bending beamline, were used. The X-rays were monochromatized by the LN_2_ cooled double-crystal monochromator. The X-ray diffraction was determined using a 4-axis Huber goniometer equipped with a BEDE-YAP detector and DECTRIS-Pilatus and Mythen detectors. The local crystal structure was examined by X-ray absorption fine structure (XAFS) measurements using a 19-element SSD detector. The chemical binding was examined by hard-X-ray photoelectron spectroscopy (HAXPES) with bulk sensitivity. The equipment consists of a Cienta R4000 spectrometer following a channel-cut monochromator and a cylindrical focusing mirror. The incident X-ray energy was calibrated to 8 keV using Au 4f signals.

### Scanning transmission electron microscopy experiments

The starting powder was dispersed in ethanol and deposited on an amorphous carbon grid supported by a Cu mesh. A piece of an NPD film specimen was cut in a cross-sectional view and mechanically polished with a diamond suspension. The sample was then dimpled using a dimple grinder. The conventional Ar ion beam thinning method was used to obtain an electron-transparent thin specimen. HAADF, LAADF and ABF STEM images were taken by a 300 kV ARM300CF electron microscope (JEOL) equipped with an aberration corrector (ETA corrector) and a cold field emission gun. The illumination half-angle was 24 mrad, and the detector angles were 12–24 mrad for ABF, 32–79 mrad for LAADF, and 79–200 mrad for HAADF imaging, where the images were simultaneously recorded.

### Simulation

The first-principles calculation of the electronic state of GaN-ZnO was performed using the commercial version of PHASE, which is a density functional theory computation software using the pseudo-potential. For the pseudo-potential, the PAW (projector augmented wave) potential was used. For the exchange-correlation interaction, the GGA (generalized gradient approximation) PBE (Perdew-Burke-Ernzerhof) functional was used. For the onsite Coulomb interaction, a Hubbard correction was made to the 3d orbitals of Ga and Zn. Ueff = 10 eV was applied to both Ga and Zn as a correction value so that the calculated position of the d band would match the HAXPES results of GaN and ZnO. The computation model for the four types of samples (GaN, Ga_0.75_Zn_0.25_N_0.75_O_0.25_, Ga_0.5_Zn_0.5_N_0.5_O_0.5_, and ZnO) was configured based on the structure of the inorganic crystal structure database (ICSD). Using the crystal structure of the ICSD and regarding the lattice constant as the tentative measured value of the XRD, the lattice constant of the minimum energy was calculated by DFT calculation while maintaining the a (=b)/c ratio, and then, the positions of the atoms were relaxed to prepare a stable structural model for each sample.

PHASE was originally developed in the RISS project supported by MEXT of the Japanese government. The original codes of the free version are currently maintained and continuously developed by a member of NIMS [https://azuma.nims.go.jp/software/phase]. Following the above activity, improved versions of those we used have been commercially distributed and are supported by multiple software venders.

### Measurement of photocurrent and flat band potential

For the measurement, a three-pole electrochemical cell was used, and a sample film (area: 0.75 cm^2^) formed on an FTO glass substrate, a platinum foil, and a Ag/AgCl (saturated KCl solution) electrode were prepared as the working electrode, the counter electrode, and the reference electrode, respectively. A 0.5 M Na_2_SO_4_ solution (pH = 6.0 to 6.5) was used for the electrolyte, and nitrogen gas was infused into the solution for 30 minutes in advance to remove dissolved oxygen. The potential value was corrected to the reference value (vs. NHE) of the standard hydrogen electrode at a pH of 0, and this value is adopted in this paper. The photoelectric current-potential curve was measured by sweeping the potential in the range from −0.4 to 1.6 V and intermittently radiating pseudo-sunlight of AM1.5G (100 mW/cm^2^) using a solar simulator.

The impedance spectrum was measured at intervals of 50 mV by applying potentials ranging from 0.4 to −0.4 V vs. NHE. The frequency range and the amplitude at each potential were 1000 kHz to 0.1 Hz and 20 mV, respectively.

The spectrum was fitted in an equivalent circuit consisting of R and C, and the electric capacitance C_sc_ of the space charge layer at each potential was calculated.

The flat band potential was calculated from the Mott-Schottky relational equation shown below.


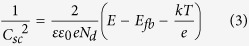


In equation 3, C_sc_ is the electric capacitance of the space charge layer, ε is the relative dielectric constant, ε_0_ is the vacuum dielectric constant, N_d_ is the donor density, E is the applied potential, E_fb_ is the flat band potential, k is the Boltzmann constant, T is the absolute temperature, and e is the elementary charge.

## Additional Information

**How to cite this article**: Imanaka, Y. *et al*. An artificial photosynthesis anode electrode composed of a nanoparticulate photocatalyst film in a visible light responsive GaN-ZnO solid solution system. *Sci. Rep.* **6**, 35593; doi: 10.1038/srep35593 (2016).

## Figures and Tables

**Table 1:** Compositions (mole ratios) of the raw material powders used for the present research.

	GaN	ZnO
GaN	1	0
GaN-ZnO solid solution	0.897	0.103
	0.758	0.242
	0.689	0.311
	0.550	0.450
ZnO	0	1

The values are the results measured in the ICP analysis.
